# The Reliability of Classification of Terminal Nodes in GUIDE Decision Tree to Predict the Nonalcoholic Fatty Liver Disease

**DOI:** 10.1155/2016/3874086

**Published:** 2016-12-07

**Authors:** Mehdi Birjandi, Seyyed Mohammad Taghi Ayatollahi, Saeedeh Pourahmad

**Affiliations:** Department of Biostatistics, School of Medicine, Shiraz University of Medical Sciences, Shiraz, Iran

## Abstract

Tree structured modeling is a data mining technique used to recursively partition a dataset into relatively homogeneous subgroups in order to make more accurate predictions on generated classes. One of the classification tree induction algorithms, GUIDE, is a nonparametric method with suitable accuracy and low bias selection, which is used for predicting binary classes based on many predictors. In this tree, evaluating the accuracy of predicted classes (terminal nodes) is clinically of special importance. For this purpose, we used GUIDE classification tree in two statuses of equal and unequal misclassification cost in order to predict nonalcoholic fatty liver disease (NAFLD), considering 30 predictors. Then, to evaluate the accuracy of predicted classes by using bootstrap method, first the classification reliability in which individuals are assigned to a unique class and next the prediction probability reliability as support for that are considered.

## 1. Introduction

Logistic regression and classification tree (CT) are two different techniques used to consider the relationship between a set of independent variables and binary response variable [1]. However, in logistic regression, by increasing the independent variables, some problems such as multicollinearity among the variables and their interactions may be serious in investigating the nature of each covariate relation. For these reasons, CT would be a suitable case for analysis of these types of variables. CT is a nonparametric method which is suitable when we aim to consider the effects of risk factors on complex diseases directly or indirectly [2, 3].

However, one of the weaknesses of classification trees is that they are very sensitive to small revision in the training set, and CT is built upon them, so that with small changes in this set the entire structure of the tree may be reformed. On the other hand, in traditional decision trees, CT analysis provides only a classification and a probability estimate. The classification results from assigning an observation to a unique class and probability estimate is the support for that classification. Due to employing this tree structure for decision-making in the clinical and applicational situation, reviewing the reliability of predicted classes is of special importance.

In order to increase the prediction accuracy and reliability of the classification tree, ensemble methods like random forests [4], bootstrap aggregation (bagging) [5, 6], and boosting [7, 8] are used. Although these methods are so suitable instruments for identifying the risk factors associated with classified response, there is no tree structure for decision-making in these methods and classes will just be determined on the basis of their majority voting [9, 10]. In studies, where CT is used for prediction and diagnosis of outcomes such as death and survival, health, and diseases based on identification of related factors, the original tree structure is usually used and the decision is done based on it. In medical research, for instance, For example, in application of CT in clinical research, we could refer to classification of heart patients according to the disease etiology [11], diagnosis of liver diseases based on its risk factors [12], detection of activity intensity in the youth with cerebral palsy [13], prediction of severe acute pancreatitis [14], and so on.

On the other hand, it would be of great worth in clinical situation if we are able to employ CT with appropriate accuracy of prediction and also be able to assess the reliability of prediction classes. The tree used in this study is the* GUIDE (Generalized, Unbiased, Interaction Detection and Estimation) tree* presented by Loh. He showed that through reducing bias selection of variables for split and employing techniques for selecting more effective variables for specified predicted class [15]. This tree* in comparison with other algorithms* like CART [3], QUEST [16], and CRUISE* [17] has a reasonable predictive accuracy and suitable depth*.

In order to evaluate the classification reliability of predicted classes few studies have been done [18]. But Kuhnert and Mengersen [19] offered a general study for measuring the reliability of terminal nodes of the CART tree, based on the bootstrap. Also, Graham et al. used this method with similar CT for assessment of local terminal nodes to improve the quality of patients care [18]. Therefore the terminal nodes with low accuracy can be identified by using Kuhnert's method on modern CT.

For this purpose, the GUIDE classification tree [15] is used in this study to recognize and predict the individuals' NAFLD based on many risk factors on training set. Then, by using the bootstrap, the samples were chosen by replacing of training set and new CTs would be formed and based on the observations of the test set the unreliable terminal nodes would be recognized. The main importance in this study is that, in addition to the modern tree with suitable accuracy used for predicting NAFLD, classification reliability and the probability of prediction of its terminal nodes (predicted classes) are evaluated. So this method can be considered as a process of development of a screening and clinical support tool, where reliability of predicted classes depends on the utility or values placed on the errors so that the researcher determines sensitivity and specificity.

## 2. Methods

### 2.1. The Dataset

The present research was a cross-sectional study conducted from January to August 2013 in Kavar city of Fars province located in the south of Iran. A total of 1600 individuals were selected randomly using cluster random sampling method from the family registration data available in Health Care Center of Kavar city and related villages.

Thirty attributes including demographic and clinical characteristics were studied to predict the binary outcome variable presenting the existence of NAFLD (with/without NAFLD). The variables used in the analysis are described in the Appendix.

Diagnosis of NAFLD was according to increased echogenicity of liver parenchyma and attenuation of portal vein or echogenicity of diaphragmatic area due to transabdominal sonography calibrated sonography machine. The sonographers were trained before the study with unique instruction [20].

### 2.2. Classification Trees

A CT is composed of root, internal, and leaf nodes. The root node is on the top of the tree and the observations are passed down the tree until they reach the internal nodes that represent a question on which a split is based. In the following step, they reach the leaf or terminal nodes which represent a classification or decision [10].

Many of the early CT algorithms, including CART [3] and C4.5 [21], by using Gini index and entropy orderly, search for a split of a node exhaustively in order to minimize the amount of node heterogeneity. As a result, if all other things were equal, variables with more values would have great chance for choosing. So, overly large or small tree structures could be produced by this bias selection and the importance of the variables would be obscure [15]. By using *F* and Chi-squared tests firstly at each node, CRUISE [17] algorithms avoid the bias in order to select the variable to split on. But a weakness of CRUISE is that the number of interaction tests is more compared to the main effect tests. As a result, CRUISE has a greater tendency to split on variables identified through interaction tests [22].

But GUIDE method, by increasing the strengths and correcting the weakness, improves upon the mentioned algorithms. In this method, when there is an interaction between *X*_*i*_ and *X*_*j*_ at a node *t*, two-level search would be used for splits. In order to yield the most reduction in impurity, firstly, the split of *t* should be found on *X*_*i*_ and the splits of its two children nodes on *X*_*j*_. Then, by reversing the roles of *X*_*i*_ and *X*_*j*_, the corresponding splits would be found. The one reducing more impurities is used to split *t*. Besides, univariate splits and bivariate linear splits of two *X* variables can be used by GUIDE at one time. The bivariate linear splits can be given higher or lower preference over univariate splits. Finally, if there was no significant interaction tests Bonferroni correction, the linear splits would be considered [23].

In this study, the whole set was then divided into a training set (almost 70% of all cases), which was used for the induction of a CT that classified the individuals into “with” or “without” risk of NAFLD, and a testing set (30%), which was used to check the accuracy of an obtained solution.

The CT was built by using NAFLD as a response variable, with the following steps: From each of the predictor variables of interest, the variable that splits the data into two groups (or nodes) with the most pure response, using prespecified criteria, was chosen. These criteria included specification of the minimum number of observations to enter each node (5 observations), the minimum number in a node before attempting to split (5 observations), and the “costs” assigned to misclassify the items. Cost is measured in terms of proportion of misclassified cases. In order to better predict the classification of patients who actually have NAFLD, different costs may be applied to the classification of the two groups [3]. For this purpose, two different structures of the tree were studied based on different misclassification costs. The first case considered equal costs for individuals with and without NAFLD and the second assigned a cost of 2 to the classification of  “high risk” so that *C*_1_ = 1 and *C*_2_ = 2. In other words, the cost of misclassification of an individual with a high risk “NAFLD” as low risk “NAFLD” is two times that of the opposite.

Allocating unequal cost and giving high weight to high risk persons increase the sensitivity of the built model in recognizing patients who really suffer from NAFLD [18]. Sensitivity is the power of a test or method to correctly classify an individual as “diseased” [24].

The resulting tree will be large and complex, so 10-fold cross-validation [15] and pruning are used to determine the best tree with the smallest cross-validated error rate.

### 2.3. Estimation of Reliability

After making the final classification tree based on GUIDE algorithm which are clinically useful, in order to identify the factors affecting the prediction of NAFLD, the method proposed by Kuhnert and Mengersen [19] was used to assess the reliability of the terminal nodes (predicted classes).

The theory used for measuring the reliability proceeds is as follows.

After splitting the data into two sets of training (70%) and test (30%), we built the GUIDE classification tree, *T*, based on training set. Then, $\hat{P}(t)$, the proportion of high risk individuals, would be estimated in each terminal node based on the test set. According to the estimated proportion and comparing it with a priori threshold “*k*,” the individuals were allocated to two classes: individuals with NAFLD $\left(\hat{C}(t)=1\right)$ and those without NAFLD $\left(\hat{C}(t)=0\right)$.

The value *k* could be considered by misclassification cost, so if this cost was considered equivalent for both those with and those without NAFLD, it would be 0.5; otherwise, the weighted average of priors probability (the proportion of patients in each class) would determine the status of the binary splits.

Now the question is “with what precision are $\hat{P}(t)$ and $\hat{C}(t)$ estimated?” In other words, the class in which the individuals are assigned as with/without NAFLD and probability supported that class, how much is it stable?

For this purpose, using the bootstrap, *B* samples were chosen by replacing of training set and new classification trees, *T*_*b*_, *b* = 1,…, *B*, would be formed by using the same criteria used in generating the original trees. Then, the observations of the test set on *T*_*b*_ trees were classified as with or without NAFLD.

Obviously, these bootstrapped trees have different structures and nodes compared to the original tree. However, these structures are not our desire, but it is important to know whether the individuals who enter from a test set to a specific node of the original tree would have the same classification in bootstrapped tree.

#### 2.3.1. Refining of Prediction Probability of Terminal Nodes

Estimated probability of success, $\hat{P}(t)$, in each terminal node of the original tree, *T*, would be refined in the following way:

(1) $$\begin{array}{c} {\hat{P}}_{B}\left(\;t\;\right)=\frac{1}{B}\overset{B}{\underset{b=1}{\sum }}{\hat{p}}_{b}\left(\;t\;\right) \end{array}$$

in which ${\hat{P}}_{b}(t)$ represents high risk individuals predicted by *T*_*b*_ in node *t* of original classification *T* by using test set.

#### 2.3.2. Classification Reliability of Terminal Nodes

The first aim of this study was to determine the reliability of terminal nodes $\hat{C}(t)$, built based on GUIDE original tree (*T*) in test set.

For this purpose, initially a probability threshold was determined to specify the class at terminal node. Then, this class was compared with the class produced based on the original GUIDE tree. The calculated estimates of the classification reliability, *R*_*c*_(*t*), show the proportion of times the bootstrap probability ${\hat{p}}_{b}(t)$ leads to the same conclusion about the classification of original tree *T*.

In other words,

(2) $$\begin{array}{c} R_{c}\left(\;t\;\right)=\frac{1}{B}\overset{B}{\underset{b=1}{\sum }}c_{b}\left(\;t\;\right)\times 100\%, \end{array}$$

where

(3) $$\begin{array}{c} c_{b}\left(\;t\;\right)=\left\{\begin{array}{cc} I\left({\hat{p}}_{b}\left(t\right)≻k\right), & \text{if }\hat{c}\left(t\right)=1,\\ I\left({\hat{p}}_{b}\left(t\right)\leq k\right), & \text{if }\hat{c}\left(t\right)=0 \end{array}\right. \end{array}$$

in which *I* represents an indicator function and *k* is the threshold.

If misclassification cost is the same for both outcomes, we will consider *k* equal to 0.5 so that if $\hat{P}(t)>0.5$ then observations will be assigned to the “with NAFLD” class and conversely they will be assigned to the “without NAFLD” class if $\hat{P}(t)\leq 0.5$. Obviously, unequal misclassification cost will change the value of this threshold through weighted average of probabilities. In fact, this is a rule used as base criteria for assigning classes to the terminal nodes in construction of the original tree.

Determining the value of *k*′ threshold for *R*_*c*_(*t*) is arbitrary and, due to the importance of the study, it can adopt different values, as the larger value indicates that the decision should be stricter. Whenever *R*_*c*_(*t*) gets closer to zero, it indicates unreliability of the corresponding class. However, we chose *k*′ equal to 0.95 in this study. In other words, a classification will be reliable if *R*_*c*_(*t*) > 0.95 and unreliable if *R*_*c*_(*t*) ≤ 0.95.

#### 2.3.3. Prediction Reliability of the Terminal Nodes

In order to estimate the prediction reliability of terminal nodes, the sampling error would be identified firstly and then a reference will be constructed. For this purpose, based on idea of Efron and Tibshirani [25], we considered the variance of the standard error of the bootstrap prediction probability in each terminal node (*se*_*B*_) of the original tree:

(4) Vse⌢BV1B∑b=1Bp^bt−p⌢Bt21/2≅μ4/μ2−μ24n2+σ2k+2n4n2B;

where $s{\overset{⌢}{e}}_{B}$ is standard error of the bootstrap prediction probability in each terminal node and *k* = *μ*_4_/*μ*_2_ − 3 represents the standardized kurtosis, *σ* ^2^ is the variance of the distribution of interest, ${\overset{⌢}{\mu }}^{2}$ and ${\overset{⌢}{\mu }}^{4}$ are second and fourth moments; with the expansion of the binomial distribution, these values are as follows:

(5) k=1−6pqnpq,μ⌢2=npq,μ⌢4=npq1+3pqn−2.

With placement of formula (4), we find

(6) Vt=2p⌢Btq^Btnt−3+14nt2︸Ι+2p⌢Btq⌢Bnt2−3+14nt2B︸ΙΙ.

As observed, *V*(*t*) is separated into two components in which *Ι* indicates sampling error and *ΙΙ* indicates resampling error which is directly caused by the bootstrap.

In (6), the large number of bootstrap samples (*B*) ensures us that the resampling error is negligible. Kuhnert and Mengersen [19] showed that if *B* = 500, resampling error would be ignored, so in this study, in order to determine the number of *B*, we considered it as 500.

For prediction reliability *R*_*P*_(*t*), *V*(*t*) should be compared with a reference *V*_*R*_(*t*).

For this purpose *V*_*R*_(*t*) should be considered so that the maximum possible variance (the worst case) could be attained. In each node *t*, bootstrap prediction would be transformed to the logit scale and multiplied by a small constant *ε*. This transformation shifts the probabilities close to 0.5, because, with this value, *V*(*t*) would have the maximum variance which may be considered as a “worst case” situation:

(7) $$\begin{array}{c} {\overset{⌢}{p}}_{b}{\left(\;t\;\right)}^{\prime }=\frac{\exp\left\{\varepsilon \log it\left({\overset{⌢}{p}}_{b}\left(t\right)\right)\right\}}{1+\exp\left\{\varepsilon \log it\left({\overset{⌢}{p}}_{b}\left(t\right)\right)\right\}}. \end{array}$$

Therefore, the maximum variance is defined as

(8) $$\begin{array}{c} V_{R}\left(\;t\;\right)\approx \frac{2{\hat{p}}_{B}{\left(t\right)}^{\prime }{\hat{q}}_{B}{\left(t\right)}^{\prime }\left(n_{t}-3\right)+1}{4n_{t}^{2}}. \end{array}$$

However, for predictions close to zero or one, more flexibility would be achieved in selecting the “worst case” by choosing of *ε*. So, in this paper *ε* = 10^−4^.

To assess the prediction reliability of the terminal nodes, Kuhnert and Mengersen [19] suggested three methods and the easiest and mostly accepted one was comparison of the observed prediction variance with the maximum variance at a terminal node:

(9) $$\begin{array}{c} R_{P}\left(\;t\;\right)=\frac{V\left(t\right)}{V_{R}\left(t\right)}. \end{array}$$

By considering *V*_*R*_(*t*) as the worst possible case, the value of *R*_*P*_(*t*) would be larger by increasing *V*(*t*) so that when *R*_*P*_(*t*) is closer to 1, it indicates unreliability of the terminal node *t*.

We can evaluate the reliability of each terminal node by comparison of *R*_*P*_(*t*) with the prespecified threshold *k*. The threshold value was considered 95% in this study so that the terminal nodes with *R*_*P*_(*t*) > 95% were unstable in terms of prediction reliability.

### 2.4. Software Used to Build Classification Tree and to Determine the Reliability of the Terminal Nodes

According to the method mentioned in the text, the package GUIDE (http://www.stat.wisc.edu/~loh/guide.html) and MATLAB 9 software were used to build the guide CT and to evaluate the reliability of the terminal nodes. In this regard, firstly the original CT in two equal and unequal cost states was built with GUIDE package based on trained data. Then, using batch program as the bootstrap, GUIDE algorithm was run. After saving the results, the reliability of classification and prediction probability of terminal nodes, according to the methodology used in the paper, were implemented by using MATLAB software.

## 3. Result

**Figure 1:**
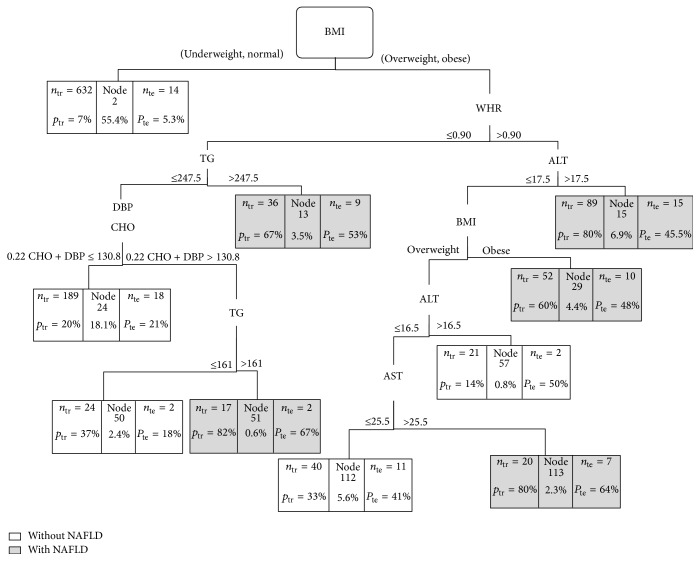
GUIDE classification tree with estimated priors probability (the proportion of patients in each class) and equal misclassification costs for predicting NAFLD. At each intermediate node, an observation goes to the left branch if and only if the condition is satisfied. Dark nodes represent predicted class “with NAFLD” and white nodes represent predicted class “without NAFLD.” Each terminal node has been formed in boxes of 3 parts so that the specified section in the left side of the box represents the number of individuals in the trained dataset who have been placed in this node. Specified percentages for this dataset are ratio of patients with NAFLD. The middle specified section of the box exhibits the node's number and specified percent below it shows the overall ratio of the test datasets which have been placed in this node. The specified section in the right side of the box shows the number and percentage of the test datasets that have really high risk of NAFLD.

**Figure 2:**
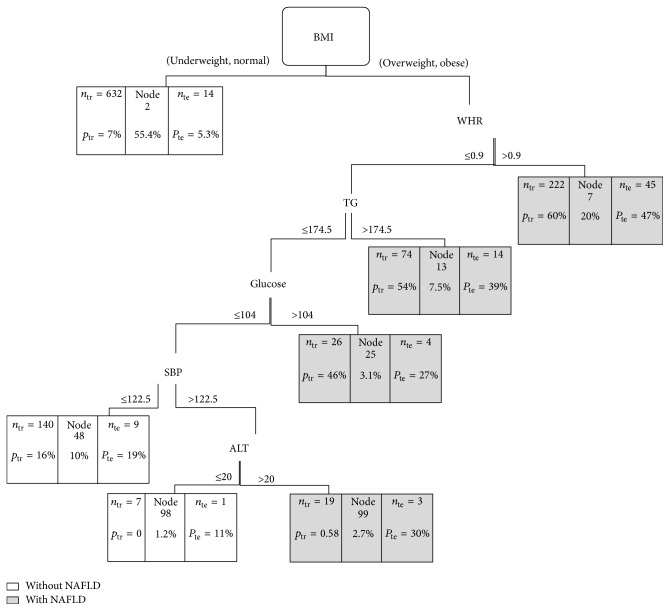
GUIDE classification tree with estimated priors probability (the proportion of patients in each class) and unequal misclassification costs for predicting NAFLD. At each intermediate node, an observation goes to the left branch if and only if the condition is satisfied. Dark nodes represent predicted class “with NAFLD” and white nodes represent predicted class “without NAFLD.” Each terminal node has been formed in boxes of 3 parts so that the specified section in the left side of the box represents the number of individuals in the trained dataset who have been placed in this node. Specified percentages for this dataset are ratio of patients with NAFLD. The middle specified section of the box exhibits the node's number and specified percent below it shows the overall ratio of the test datasets which have been placed in this node. The specified section in the right side of the box shows the number and percentage of the test datasets that have really high risk of NAFLD.

1600 individuals participated in this study, among whom 1120 were placed in trained dataset in order to build the CT and 480 were placed in test dataset in order to evaluate it. 30 predictor variables were used in the construction of the CT to predict the risk of NAFLD. The built trees were analyzed in two equal and unequal misclassification cost states. The obtained results are shown in Figures 1 and 2.

### 3.1. Classification Tree with Equal Misclassification Costs

Figure 1 illustrates the CT made with respect to equal misclassification cost. The CT has 10 final nodes where each terminal node indicates the classification of having or not having NAFLD. Regarding the complex interaction between independent variables, this CT can be useful in predicting the risk of NAFLD.

For example, BMI at the top of the CT indicates that this predictor is the most influential factor in NAFLD. In obese and overweight people, if WHR is more than 9.0 and ALT is more than 17.5, the probability of NAFLD is 80% and the predicted class is “with NAFLD.” But the interesting issue about this tree which is not usually observed in different CT algorithms is the part which uses a linear combination of two variables for prediction. As Figure 1 shows, among obese and overweight people whose WHR is less than 9.0 and whose triglyceride level is less than or equal to 247.5, while 0.22 CHO + DBP ≤ 130.8, then the risk of NAFLD is 20% and the predicted classification is “without NAFLD.” Otherwise, the process continues in accordance with the figure.

To test the validity of the trained CT, the test dataset was used in the CT.  Table 1 shows a cross tabulation of the observed and predicted NAFLD of the CT for trained and test samples.

The diagnostic accuracy of the original CT based on trained and test datasets was 85% and 81%, respectively. Moreover, according to the trained dataset, the sensitivity and specificity were 59% and 93%, respectively. These values were 48% and 89% in test datasets. Although the specificity and accuracy of the CT overall prediction are good based on these two datasets, the diagnosis of this CT is not suitable for those who really suffer from NAFLD.

### 3.2. Classification Tree with Unequal Costs

Figure 2 illustrates the CT made with unequal costs based on trained dataset. In this tree, the cost of misclassification of an individual with a high risk “NAFLD” as low risk “NAFLD” is two times that of the opposite.

This CT has 7 final nodes and it is similar to Figure 1 in which BMI and WHR, as the most important factors in the diagnosis of NAFLD, are at the top of the CT, but the order of later predictors is somewhat different so that the probability of having NAFLD among obese individuals with WHR more than 0.9 is 60% and the predicted class is “with NAFLD.” Among obese people with WHR less than 0.9, triglyceride will be the next predictor and the next stages are shown in Figure 2.

Contingency Table 2 shows that the accuracy of diagnosis based on trained and test datasets is 81% and 75%, respectively, and the sensitivity and specificity of the CT according to trained dataset are 74% and 83%, respectively, while on the basis of test dataset they are 73% and 76%, respectively.

As shown in Table 2, with a slight decrease in the total accuracy of the CT, the sensitivity increases considerably. In other words, this CT can better identify the patients with high risk of NAFLD.

### 3.3. Reliability of Classification Tree with Equal and Unequal Costs

In order to assess the reliability of the terminal nodes of the CT in terms of prediction probability and its corresponding classes, the methodology described in Section 2.3 was applied. The results of CT with equal misclassification cost are given in Table 3.

As seen, nodes 51, 13, and 113 are unstable nodes. Node 51 is highly unreliable, in terms of both classification and prediction probability. Analyzing this node, we can observe that 3 patients of the test dataset are in this node. Low number of samples in this node can be interpreted as a reason for its being “unreliable.” Nodes 13 and 113 represent a different rate of reliability so that both are unreliable in terms of classification, but they are not unreliable in terms of probability of prediction.

However, *R*_*p*_(*t*) of these two nodes reveals that these values are less than 0.95 but the difference is very slight. In other words, more caution is required in reporting the accuracy and reliability of these two nodes.

Moreover, results of Table 4 show the reliability of the terminal nodes for CT with unequal misclassification costs (Figure 2). As seen, node 7 is unreliable in terms of classification, but it is reliable in terms of prediction. Anyway, its *R*_*p*_(*t*) value is near the threshold (95%) and we should use it cautiously for interpretation of NAFLD prediction. In this CT, we cannot predict NAFLD based on BMI and WHR only and it may be the reason for its unreliability. So, for its prediction, recognition of more risk factors is needed.

As seen, the remarkable issue in this paper is that unreliable nodes all belong to classes which predict individuals with NAFLD.

## 4. Discussion

The obtained results based on both trees are acceptable from clinical point of view so that, according to the conducted studies based on univariate analysis, the selected variables for constructing a tree are introduced as risk factors for NAFLD. For example, like almost all studies in the field of NAFLD, BMI has been introduced as the main risk factor for catching and predicting NAFLD [26–29].

Similarly, in many studies waist circumference has been shown as another risk factor for NAFLD [26, 27] while in a study done by Ahad Eshraghian et al. [30] it has been shown that WHR is a more accurate risk factor for NAFLD in comparison to waist circumference, where it was introduced as a risk factor for making tree in this study.

Also, Bedongi et al. [31] showed that BMI, high TG, and waist circumference are the three important risk factors in diagnosing NAFLD. Our results showed these three risk factors are the main ones and at the top of the tree.

Four risk factors including high SBP, WHR, BMI, and high TG are the determiners of NAFLD, part of metabolic and anthropometric features of metabolic syndrome [32]. So there is a close relationship between NAFLD and metabolic risk factors. Interestingly, these metabolic risk factors have been diagnosed in such a tree with high sensitivity (Figure 2) and they could diagnose those who really suffer from NAFLD. Other variables like ALT and glucose that were diagnosed in both trees have been introduced as risk factor for NAFLD in different studies [33–35]. Anyway, the advantage of these trees, in comparison with univariate analysis, is that interactions between dependent variables were considered too.

Based on the obtained results in the present study, equal misclassification costs in CT lead to a higher total accuracy compared with unequal costs. Indeed, the accuracy of prediction for the individuals with higher risk or with “NAFLD” is relatively low whereas a CT with unequal costs can considerably better predict patients with “NAFLD.” In other word, this tree is more sensitive. In diagnosing diseases particularly when late diagnoses has irreparable consequences the power of diagnostic test is of great importance. So long as the sensitivity of a test increases its power in diagnosing real patients will increase. For example, in classification tree (Figure 1) the sensitivity of the training test is 48% and it reaches 73% in Figure 2 with unequal cost. It means that through tree Figure 1 we are able to detect only 48% of those who are really NAFLD infected while via tree Figure 2 this ability increased to 73%. Anyway, proportional to the variation of cost of incorrect classification we could construct a tree with high sensitivity or specificity regarding the importance of our study.

The misclassification error rates in both test and trained datasets for both CTs with equal and unequal costs are relatively close together. However, it is expected that the method built based on trained dataset reports fewer errors compared with the test dataset due to overfitting.

On the other hand, using reliability methods allows us to recognize suitable diagnosis tools for predicting NAFLD based on it. Individuals who enter unreliable nodes may need to be more carefully monitored because there may be other factors associated with the diseases which have not been collected yet. If the focus is placed on the correct prediction of risk NAFLD, analyses can reveal why there is insufficient accuracy at these nodes.

Results of this study suggest the possibility of the other independent variables needed to identify certain subgroups of patients or further data needed for classification of subgroups.

The interesting point in this study was that in some cases, for equal misclassification cost, the unreliable prediction probabilities could produce stable classifications so that, even in a case in which the classification was unstable, the prediction probability with regard to the specified threshold value was stable. *R*_*p*_(*t*) is high and close to 95% and it shows that in these cases cautious decisions should be adopted.

It is also notable that the reliabilities of classification and prediction probability were measured based on their dependence on the threshold value in this study. Because different interpretations of results are made by changing this value, it would depend on decision-maker's intention and the design of the study. Basically, the more the threshold value is, the tougher decision-making would be on the classification validity.

One of the objectives of this study was to assess the reliability of prediction in regard to the variance of the standard error of the bootstrap probability. Also, Kuhnert and Mengersen [19] used the standard error of prediction $\text{Se}(\hat{P}(B(t)))$, itself. Although in this study we could obtain the right value of ${\hat{P}}_{B}(t)=0.5$ by considering *ε* = 10^−4^, <one of the advantages of $\text{Se}(\hat{P}(B(t))$ is that if the selected *ε*, ${\hat{P}}_{B}(t)\neq 0.5$, the equality hypothesis of ${\hat{P}}_{B}(t)$ with 0.5 using *t*-test can be studied.

One limitation of this study was the small sample size. Naturally, if the sample size was larger, it would be possible to build a more accurate tree. In addition, more individuals could be assigned to the test group and the reliability of the terminal nodes could be better predicted. Of course, it seems that if the prevalence of the disease is to be further investigated, the number of individuals with NAFLD in subgroups would also be larger and therefore the reliability of the terminal nodes could be estimated more accurately.

As one of the most useful features of the CT is the adaptability of missing data through identifying alternative splits, more comprehensive studies will be useful to investigate the prediction and classification reliability of terminal nodes with regard to the missing data.

This study used “GUIDE” CT to predict NAFLD. Anyway, using different classification trees (e.g., CART, CRUST) and comparing the accuracy, the reliability, and prediction's probability of terminal nodes based on the mentioned method could be useful.

One of the limitations of this study is that the variables involved acute hepatitis and heart failure and autoimmune causes were not considered, and by adding them to the structure of the tree the value of the paper would be increased. It is suggestion that a general tree by regarding all risk factors is being made and the classification reliability and the probability of prediction are considered.

Furthermore, the CT can be built by considering different grades of NAFLD as a response by using misclassification costs with ordinal response and the accuracy and reliability of their terminal nodes can be studied.

Also it is suggested that jackknife resampling method be used in order to study the reliability of the terminal nodes of the CTs made based on training sets and the reliability of these nodes would be considered by a similar method.

## 5. Conclusion

The CT with unequal misclassification costs had higher accuracy for recognizing individuals with NAFLD. Also the predicted classes were more reliable. The final result is that the low number of observations in terminal nodes of CT increased the probability of node's unreliability. So the decision for predicted classes should be done more cautiously.

## Figures and Tables

**Table 1:** Cross tabulation of the observed and predicted NAFLD of the classification tree for training and test sample and the measures of evaluating the classification tree with equal misclassification cost.

Observed	Predicted					
	Training sample			Test sample		
	Yes	No	Total	Yes	No	Total
Yes	157 (59%)	111 (41%)	268	43 (48%)	47 (52%)	90
No	58 (7%)	794 (93%)	852	42 (11%)	348 (89%)	390
Total	215	905	1120	85	395	480

*Diagnosis accuracy*	85%			81%		

**Table 2:** Cross tabulation of the observed and predicted NAFLD of the classification tree for training and test sample unequal misclassification cost.

Observed	Predicted					
	Training sample			Test sample		
	Yes	No	Total	Yes	No	Total
Yes	197 (74%)	71 (26%)	268	66 (73%)	24 (27%)	90
No	144 (17%)	708 (83%)	852	94 (24%)	296 (76%)	390
Total	341	779	1120	160	320	480

*Diagnosis accuracy*	81%			75%		

**Table 3:** Result from bootstrapping the classification tree with equal costs for classification and prediction reliability of terminal nodes.

Node *t*	Information ${\hat{P}}_{B}(t)$	Classification reliability *R*_*C*_(*t*)%	Prediction reliability *R*_*P*_(*t*)%
2	0.003	100	1.75
13	0.339	**13.4**	90.88
15	0.334	100	89.64
24	0.057	100	23.3
29	0.296	100	85.06
50	0.057	100	37.28
51	0.3	**27.2**	**100**
57	0.125	100	81.2
112	0.194	100	65.34
113	0.353	**21**	93.13

Unreliable nodes (those with classification reliability less than 95 percent or prediction reliability more than 95 per cent) are in bold font.

**Table 4:** Result from bootstrapping the classification tree with unequal costs for classification and prediction reliability of terminal nodes.

Node *t*	Information ${\hat{P}}_{B}(t)$	Classification reliability *R*_*C*_(*t*)%	Prediction reliability *R*_*P*_(*t*)%
2	0.006	100	3.17
7	0.353	**14.4**	91.55
13	0.281	100	81.92
25	0.153	100	58.67
48	0.075	100	30.82
98	0.041	100	36.83
99	0.168	100	34.29

Unreliable nodes (those with classification reliability less than 95 percent or prediction reliability more than 95 per cent) are in bold font.

**Table 5:** Table of demographic and clinical characteristics of participants according to groups (number (%) or mean ± SD).

Risk factors	Abbreviation	Level	Without NAFLD (*n* = 1241)	With NAFLD (*n* = 359)
Sex	SEX	Male	361 (% 29.1)	110 (% 30.7)
		Female	880 (% 70.9)	249 (% 69.3)
Marital status	MS	Single	447 (36%)	27 (7.5%)
		Married	726 (58.5%)	297 (83%)
		Other	68 (5.5%)	35 (9.5%)
History of hepatitis B vaccine	HEP	Yes	538 (43.4%)	70 (19.3%)
		No	703 (56.6%)	289 (80.7%)
History of blood transfusion	BT	Yes	22 (1.8%)	11 (3.1%)
		No	1219 (98.2%)	348 (96.9%)
Thalassemia	THAL	Yes	2 (.2%)	1 (.3%)
		No	1239 (99.8%)	358 (99.7%)
Hemophilia	HEMO	Yes	3 (.2%)	0 (.0%)
		No	1238 (99.8%)	359 (100%)
Dialysis	DI	Yes	3 (.2%)	1 (.3%)
		No	1238 (99.8%)	358 (99.7%)
Surgery	SU	Yes	3 (.2%)	1 (.3%)
		No	1238 (99.8%)	358 (99.7%)
History of surgery	HS	Yes	356 (28.7%)	141 (39.4%)
		No	885 (71.3%)	218 (60.4%)
History of dental surgery	DE	Yes	1002 (80.7%)	303 (84.6%)
		No	239 (19.3%)	56 (15.4%)
History of phlebotomy	PH	Yes	94 (7.6%)	35 (9.8%)
		No	1147 (92.4%)	324 (90.2%)
Tattoos	TA	Yes	38 (3.1%)	19 (5.3%)
		No	1203 (96.9%)	340 (94.7%)
History of unsanitary piercing ears	UPE	Yes	541 (43.6%)	141 (39.4%)
		No	700 (56.4%)	218 (60.6%)
Hookah	HOO	Yes	83 (6.7%)	28 (7.8%)
		No	1158 (93.3%)	331 (92.2%)
Current smoking	SMOK	Yes	39 (3.1%)	19 (5.3%)
		No	1202 (96.9%)	340 (94.7%)
History of drug using	HDU	Yes	28 (2.3%)	6 (1.7%)
		No	1213 (97.7%)	353 (98.3%)
HBS Ag	HBSAG	Negative	1215 (98.1%)	353 (98.5%)
		Positive	26 (1.9%)	6 (1.5%)
HBS Ab	HBSAB	Negative	1079 (88.5%)	307 (87.0%)
		Positive	162 (11.5%)	52 (13.0%)
Body mass index	BMI	Underweight (UW)	197 (15.9%)	1 (.3%)
		Normal (N)	633 (51%)	62 (17.3%)
		Overweight (OW)	320 (25.8%)	186 (51.7%)
		Obese (OB)	87 (7%)	110 (30.7%)
Waist-hip ratio	WHR		0.83 ± 0.09	0.92 ± 0.09
Systolic blood pressure	SBP		100.05 ± 26.1	108.42 ± 31.86
Diastolic blood pressure	DBP		82.14 ± 20.01	93.37 ± 23.85
High density lipoprotein	HDL		50.95 ± 11.5	48.9 ± 9.73
Triglycerides	TG		120.3 ± 68.52	193.89 ± 113.5
Alanine aminotransferase	ALT		15.56 ± 10.92	19.11 ± 12.5
Cholesterol	CHO		184.94 ± 42.58	207.62 ± 41.79
Aspartate aminotransferase	AST		24.84 ± 11.66	28.06 ± 17.84
Glucose	GLU		96.68 ± 26.86	108.45 ± 39.56
Albumin	AL		4.32 ± 0.37	4.23 ± 0.4
Age	AGE		34.85 ± 17.45	45.9 ± 13.34

## Appendix

See Table 5.
